# Implementing cognitive therapies into routine psychosis care: organisational foundations

**DOI:** 10.1186/s12913-015-0953-6

**Published:** 2015-08-05

**Authors:** Frances Dark, Harvey Whiteford, Neal M. Ashkanasy, Carol Harvey, David Crompton, Ellie Newman

**Affiliations:** School Public Health, The University of Queensland, Brisbane, 4072 Australia; Business School, The University of Queensland, Brisbane, 4072 Australia; Department of Psychosocial Research, The University of Melbourne, Melbourne, Australia; Metro South Mental Health District, Sanders Street, Upper Mt Gravatt, QLD Australia; Metro South Health District, 519 Kessels Road, Macgregor, QLD 4109 Australia

**Keywords:** Mental health, Mental health services, Cognitive behavioural therapy, Cognitive remediation, Evidenced based mental health care, Psychosis

## Abstract

**Background:**

Treatment outcomes for people diagnosed with psychosis remain suboptimal due in part to the limited systematic application of evidence based practice (Adm Policy Ment Health, 36: 1-7, 2009) [1]. The Implementation science literature identifies a number of factors organisationally that need to be considered when planning to introduce a particular EBP. Profiling these organisational characteristics at baseline, prior to commencement of service reform can determine the focus of a subsequent implementation plan. This study examined the organisational baseline factors existing in two services promoting the routine use of cognitive interventions for psychosis. One of the services studied has since undertaken organisational structural reform to facilitate the greater uptake of Evidence Based Practice (EBP). The results of this study were used to design an implementation strategy to make cognitive therapies a part of routine psychosis care.

**Methods:**

One hundred-and-six mental health staff from two metropolitan mental health services in Australia was surveyed to ascertain their attitudes, competencies and interest in Cognitive Behavioural Therapy for psychosis (CBTp) and Cognitive Remediation Therapy (CRT). In addition perceptions of organisational values were profiled using the Organisational Culture Profile (OCP). Fifty five participants were excluded because they completed less than 50 % of the survey. The final sample consisted of 51 participants.

**Results:**

48.1 % of surveys were completed. Over 50 % of staff were interested in CBTp and CRT approaches to psychosis. Staff were aware of existing CBTp and CRT programs but these were not uniformly available throughout the services. Fourteen percent of staff identified as CBT therapist and 35 % were trained CRT facilitators. Only 12 % of staff were receiving therapy specific supervision. The Organisational Culture Profile (OCP) at baseline revealed highest scores amongst leadership, planning, and humanistic workplace domains, with communication receiving the lowest rating indicative of organisational weakness.

**Conclusion:**

Profiling the factors associated with successful implementation of service reform informed the implementation planning and the efficient deployment of resources in a mental health service introducing cognitive therapies for psychosis into routine clinical care. The majority of staff had positive attitudes to the evidence based cognitive therapies allowing a focus on training and supervision and the development of supporting organisational elements.

## Background

It is widely accepted that comprehensive psychosis care involves the combination of psychopharmacology and psychosocial interventions. While clinical practice guidelines related to optimal medication for psychosis have generally had reasonable implementation, it is widely recognized that effective and safe psychosocial interventions such as Cognitive Behavioural Therapy (CBT) and Cognitive Remediation Therapy (CRT) are less widely implemented [2].

A review of the implementation evaluation literature found that treatment guidelines, policies and training are ineffective on their own [3]. Multilevel implementation strategies are recommended with attention given to the stages of implementation (exploration and adoption, program installation, initial implementation, full adoption and maintenance) as well as a focus on implementation drivers (competency and capability of staff, organisational characteristics and leadership). The Consolidated Framework for Implementation Research (CFIR) provides an overarching structure to aid the systematic consideration of the complexity involved. The CFIR consists of five major domains (intervention characteristics, outer context, inner context, characteristics of the people involved and the implementation process) with further particular constructs attached to each domain [4].

### Implementation and dissemination of evidenced based mental health practices

Implementation research, which focuses on how best to engage clinicians in changing their behaviour and to build service platforms that respond to the changing landscape of evidence-based medicine, is a relatively under-developed field in mental health services.

Complex innovations such as the implementation of psychosocial interventions in mental health systems depend on changes in clinician behaviour [4, 5]. There are two common barriers to the use of Evidence Based Practice (EBP) by mental health staff reported in the literature; (a) lack of knowledge and skill; and (b) organisational dynamics that discourage innovative practice [6]. Up-skilling staff involves attention not only to training, but coaching and supervision. In Australia skill competencies are assessed in a structured way at university level before practitioners enter mental health service. However, in-service training in the work place can be less systematic and not translate into improved practice due to organisational factors such as lack of quarantined therapy and supervision time as well as individual staff issues such as retention [7].

Developments in organisational psychology and management have been introduced into mental health systems with leadership training, quality management and interactive staff training [6]. These strategies alone have not realized the routine use of EBP. There are examples of successful innovative implementation of EBP in mental health, notably Assertive Care Teams, Early Psychosis services and Dialectical Behaviour Therapy [7, 8]. These programs share common elements which include; specific treatment components; accountability systems for staff and organisations; specification of the program in relation to organisational context as well as staff case weightings to enable quarantine therapy time, training and supervision and iterative quality improvement [9]

The relationship of organisational culture to organisational performance is of particular interest in this research as organisational culture may act as a covert barrier to realising the improvements envisaged by the organisational restructure. Organisational culture in essence refers to the core, embedded values of the organisation and encompasses individual or group ways of processing information within the organisation that is often passed onto new members [10]. The relationship of organisational culture to effective practice in healthcare requires further research [11]. What evidence exists suggests that effective organisations have a culture supporting staff and are responsive to change to improve practice [11].

Organisationally the need for innovation and change needs to be well articulated and accepted by multiple stakeholders at multiple levels (consumer, staff, service, and state government) [3]. In regards to psychosis care the need to improve outcomes for people living with schizophrenia is widely accepted with different motivations from personal lived experience, to the health professional delivering care, to socioeconomic concerns about the cost of psychosis [12].

### Inner (service) context

The Brisbane Mental Health Service (MHS) planning to restructure, Metro South serves a population of around 920,000 of which three thousand people have severe and persistent mental illness with complex needs. The area has high rates of socioeconomic disadvantage with 19.8 % of people postponing mental health care because of cost. The proposed comparison service, Metro North had a similar demographic profile with a population of around 900,000. The geographic areas covered inner metropolitan suburbs to semi-rural areas in both service districts [13].

In 2012 the Metro South MHS reorganised along diagnostic/model of service lines. Seven Academic Clinical Unit’s (ACU) were established; Rehabilitation, Psychosis, Mood, Aged care, Consultation/Liaison, Resource and Access and Child and Youth. The model was influenced by a similar structure in Kings College Health service in London. This was an innovative organisational change aimed at addressing systemic issues in quality of service provision and optimal resource allocation. The service was the only MHS in Queensland to undertake this reform. One aim of this restructure was to facilitate the adoption of EBP and therapies in mental health.

At the time of the organisational change there were four CRT programs and four group CBTp derived programs all operating in the service catchment area. The aim of implementation was to upscale these therapies to enable accessibility to all consumers needing and wanting these interventions and to embed the programs within the organisational plan to promote program quality and sustainability. The first author had provided CRT training on four occasions to 36 staff and training in a form of CBTp, Social Cognitive Interaction Training (SCIT) on two occasions to 32 in 2013.

### External context

A number of potentially favourable factors were operative in the external organisational context with the Queensland Mental Health Plan (2007–2017) emphasising five key priorities; promotion, prevention and early intervention, integrating and improving the care system, participation in the community, co-ordinating care and workforce, information quality and safety [13].

Apposite to this, the restructure of the service was undertaken at a time of unprecedented budget restraint with no funding assigned for this change process [14]. In addition the restructure occurred within a broader “external context” change from centralised control of health services to local area health networks.

### Study aims and research questions

The broad study aim was to detail the potentially modifiable, baseline “inner context variables” existing at the time of organisational change. This information will inform the future implementation strategy for CBTp and CRT. Subsequent studies will evaluate the implementation outcomes in the restructured service and compare with a similar service that had interest in these therapies but had not undertaken organisational structure reform.

#### Research questions

1. What are the baseline staff attitudes, capability and competencies in implementing CBTp and CRT prior to implementation of these therapies?
2. What is the organisational culture profile in the services studied?

## Method

### Participants

One-hundred-and-six clinical staff members were recruited from community mental health teams of two large mental health services in Brisbane, Australia. Fifty five participants were excluded from the analysis because they completed less than 50 % of the survey. The final sample consisted on 51 participants. The sample consisted of 73 % females, and 27 % males. The mean age of the sample was 40 years (SD = 9.2). The mean years of working within the organisation was 7.4 years (SD = 6.6), with a range of 3 months to 33 years.

Thirty nine percent were nursing staff, 18 % medical staff, 13 % occupational therapists, 15 % social workers and 15 % psychologists. The staff were also asked their major role with 51 % identifying as case managers, 9 % psychologists, 4 % psychiatrists, 14 % training psychiatry registrars, 6 % nurses, 8 % team leaders and 8 % not specifying their role.

### Measures

The survey assessed current level of training, supervision, and interest in CBTp and CRT. The survey included a measure of organisational culture, the Organisational Culture Profile (OCP) [15, 16]. A review of organisational culture measures in health care advises that the choice of instrument should be influenced by the purpose of the study and availability of resources [17]. The OCP was chosen to be used primarily as a descriptive, formative tool that could profile the relative organisational cultural strengths and weaknesses existing during the implementation period. The OCP had been developed in Queensland by one of the authors and the dimensions had good face validity to track trends in key dimensions of organisational culture over the 3 years of the study. The OCP is a 65 item questionnaire to measure organisational culture tapping into 10 domains; leadership, structure, innovation, job performance, planning, communication, environment, humanistic workplace, development of the individual and socialisation on entry [10]. The instrument was developed after analysis of all dimensions used in 18 surveys of organisational culture resulting in 10 summary dimensions. It is constructed using a 7 point Likert scale with 1 being “strong disagreement” and 7 being” strong agreement”. Analysis of the psychometric properties of the OCP in the Australian regional healthcare sector found the domains of leadership, planning, communication and humanistic workplace as reliable dimensions (Cronbach alpha >0.80). Environment, job performance, and development of the individual reached acceptable reliability (Cronbach alpha >0.70). OCP validity was found with significant correlations between r = .67 – r = .79 [18]. A factor analysis found that 2 factors accounted for the data but the authors believe the 10 factors are more useful and descriptive [15].

### Ethics

Ethical approval for the study was obtained from the Metro South Health Service District and Metro North Health Service District Human Research Ethics Committees. Participants were provided with an information letter and consent form prior to commencement of the study.

### Statistical analyses

Data was analysed using Statistical Package for the Social Sciences (SPSS) V20. Variables were screened for entering errors, range errors, and outliers. Surveys with less than 50 % completed data were excluded from the study. Univariate statistics were carried out on the proportions of respondents who endorsed various items on the OCP and Staff Survey. Mean and Standard Deviations were calculated for the 10 subscales and total commitment score of the OCP.

## Results

**Fig. 1:**
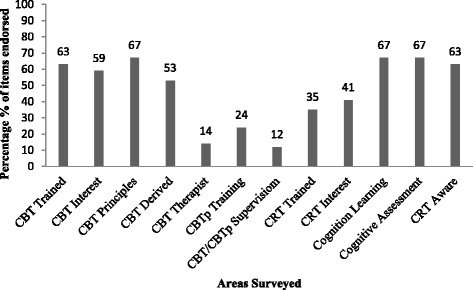
Staff CBT/CRT survey

There were 51 complete responses from 106 surveys, constituting an 48.1 % completion rate. The survey found 59 % of staff were interested in CBT and 41 % interested in CRT. CBT principles were reported as being used routinely by 67 % of staff. Another 14 % of the sample identified themselves as CBT therapists having received formal undergraduate or postgraduate training. Therapy specific supervision was accessed by 12 % of staff. Twenty-four percent of staff have had specific training in CBTp. Thirty-five percent of staff were already trained and working as CRT facilitators with a further 41 % of staff interested in training in CRT to become program facilitators. The majority of staff indicated they were interested to learn more about cognition and cognitive assessment in psychosis (67 %) (Fig 1).

**Fig. 2:**
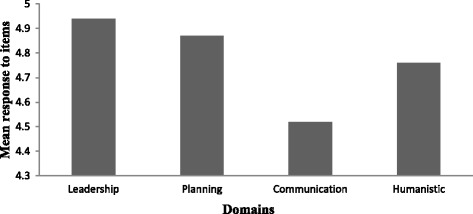
Four reliable dimensions of the OCP

The four reliable dimensions of the OCP were assessed with the highest scores across leadership, planning, and humanistic workplace dimensions. The lowest rating indicative of weakness in organisational culture was communication. The range of domains scores was between 1 and 7, the average rating was 4.8, with 4 being “undecided or irrelevant to you and 5 being undecided but inclined to agree” (Fig 2 and Table 1).

**Table 1:** Mean (M) and Standard Deviation (SD) of Organisational Domains and overall Commitment scores across Metro South *N* = 87

	M	SD
Leadership	4.9	1.23
Planning	4.87	1.10
Communication	4,52	1.24
Humanistic	4.76	1.27

## Discussion

The aim of this study was to profile the potentially modifiable inner context variables of staff attitudes, capabilities and competencies in CRT and CBTp prior to organisational restructure which would facilitate a service wide implementation plan for these therapies. The Organisational Culture Profile was used to describe potentially more covert staff attitudes that may influence implementation over the subsequent course of service change. From the literature it was predicted that the organisational culture and a lack of knowledge or perceived skills in these therapies would adversely influence staff attitudes to the implementation of CRT and CBTp [19]. The staff survey identified a number of facilitating factors within the services studied; an interest in the therapies in question and a number of staff interested in enhancing their skills to become CRT and CBTp therapists. In addition the organisational culture was perceived as relatively strong in leadership, planning and humanistic workplace dimensions. Effective organisations tend to be supportive of their employees and receptive to change and innovation [6]. In a qualitative cross case analysis of two medium sized Australian general hospitals there was a strong association between organisational culture, communication and quality care [18]. Different domains of culture are thought to be more relevant at the various stages of implementation, with strong leadership linked in the literature to facilitating change processes and more positive attitudes of staff to EBP [19]. It will be necessary to track organisational culture over the phases of implementation. In particular the organisational structure reform planned for Metro South may enhance the current assessment by staff of the innovative culture of this service.

### Limitations

The survey was paper based which likely influenced the response rate and missing data. The principle investigator also trained in the therapies studied which could have resulted in inflated scores. The staff survey questions were designed for this study to specifically ask about the therapies of interest but was not standardised.

### Strengths

The study adds to the small but growing recognition of how implementation research can contribute to systematic service reform in mental health.

### Implications for future service development

Internal factors such as service structure and workforce are malleable and under more direct local control compared with external variables such as political health policies and overall government health funding. This study demonstrated interest by staff in CBTp and CRT but a need to focus on training and therapy specific supervision to ensure competent practice. Supervision, consultation and coaching in a new practice have been identified as essential adjuncts to training and facilitating new skill use [3]. Postgraduate training will be required to enable the dissemination of these therapies. The cost of this training needs to be accounted for. The lead author of this study trains staff in both therapies for no extra cost to the service. To sustain these programs training for trainers will be required with appropriate credentialing.

The dissemination of EBP was a key platform of the organisational transformation in Metro South MHS. This has permitted the middle managers to prioritise up skilling of their staff. Endorsement of this training needs to continue beyond the initial implementation to the adoption and maintenance phase of implementation. These programs were the initiative of staff and received little if any funding and passive organisational support without a strategic direction and plan. Future research will describe the impact of a strategic implementation plan for cognitive therapies and audit the distribution and reach of these evidenced based programs to assess equity of access across the large geographic district of the study service.

### Implications for future Implementation and dissemination

This study profiles the baseline internal variables that will influence the implementation of CBTp and CRT within the Metro South health district. In this study staff reported valuing these therapies, and the organisational plan to restructure is supportive of EBP generally. The plan will focus on the supporting organisational sub-components of the implementation of cognitive therapies for psychosis that include the roles of an implementation team and governance committee (The Therapies Oversight Committee - TOC) to monitor fidelity of practice and staff competency. To this end the Rehabilitation and Psychosis ACU’s established an implementation team, Psychological Therapies for Psychosis (PTFP). This committee has developed a communication plan to ensure dissemination of knowledge about the programs to clinicians and teams and to executive. Enhancing communication is particularly relevant as at baseline on the OCP this was a weak domain in the organisational culture. The PTFP group has developed an implementation strategy; a psychological therapy algorithm and will co-ordinate the running, supervision and evaluation of therapies initiated. This committee will oversee regular program audits and fidelity checks. The group reports to the Therapies Oversight Committee and the Clinical Directors of the ACU’s and then to the executive of the service as part of a multilevel implementation strategy. The TOC advocates to the service executive about funding requirements for the programs including support for training. Further research will examine the maintenance, quality and reach of CBTp and CRT programs within the service. Metro North mental health service will be a comparison site where staff also valued cognitive therapies for psychosis but there was no strategic implementation plan focussed on these therapies.

## Conclusion

The underlying premise of this study was that access to the key evidenced based therapies for psychosis, CBTp and CRT, should be available routinely. Applying knowledge from the implementation literature can enable this to occur within facilitating services. The constrained funding environment of health services has put a greater emphasis on planning to enable the efficient use of resources. In this study surveying the baseline variables known to influence implementation of innovative practice assisted in the efficient planning for service reform to enable the embedding of cognitive therapies within routine psychosis care.

## Abbreviations

ACU: Academic Clinical Unit
CBT: Cognitive Behavioural Therapy
CBTp: Cognitive Behaviour therapy for psychosis
CRT: Cognitive Remediation Therapy
EBP: Evidence Based Practice
HHS: Health and Hospital Service
NEAR: Neuropsychological Educational Approach to Cognitive Remediation
OCP: Organisational Culture Profile
RBWH: Royal Brisbane and Women’s Hospital
SCIT: Social Cognitive Interaction Training
SD: Standard Deviation
TPCH: The Prince Charles Hospital
